# COVID-19 im Alter – Die geriatrische Perspektive

**DOI:** 10.1007/s00391-021-01864-0

**Published:** 2021-02-17

**Authors:** R. Wirth, C. Becker, M. Djukic, C. Drebenstedt, H. J. Heppner, A. H. Jacobs, M. Meisel, G. Michels, R. Nau, J. Pantel, J. M. Bauer

**Affiliations:** 1 Deutsche Gesellschaft für Geriatrie (DGG), Berlin, Deutschland; 2 Klinik für Altersmedizin und Frührehabilitation, Marien Hospital Herne – Universitätsklinikum der Ruhr-Universität Bochum, Hölkeskampring 40, 44625 Herne, Deutschland; 3 Klinik für Geriatrie, Robert-Bosch-Krankenhaus Stuttgart, Stuttgart, Deutschland; 4 Geriatrisches Zentrum, Evangelisches Krankenhaus Göttingen-Weende, Göttingen, Deutschland; 5 Abteilung für Neuropathologie, Universitätsmedizin Göttingen, Göttingen, Deutschland; 6 Klinik für Innere Medizin und Geriatrie, St.-Marien-Hospital Friesoythe, Friesoythe, Deutschland; 7 Klinik für Geriatrie, Helios Klinikum Schwelm, Lehrstuhl für Geriatrie, Universität Witten-Herdecke, Schwelm, Deutschland; 8 Klinik für Geriatrie mit Neurologie, Johanniter Krankenhaus Bonn, Bonn, Deutschland; 9 CIO, Rheinische Friedrich-Wilhelms-Universität Bonn, Bonn, Deutschland; 10 EIMI, Westfälische Wilhelms-Universität Münster, Münster, Deutschland; 11 Klinik für Innere Medizin und Geriatrie, Diakonissenkrankenhaus Dessau, Dessau, Deutschland; 12 Klinik für Akut- und Notfallmedizin, St.-Antonius-Hospital gGmbH Eschweiler, Akademisches Lehrkrankenhaus der RWTH Aachen, Eschweiler, Deutschland; 13 Institut für Allgemeinmedizin, Johann Wolfgang Goethe-Universität Frankfurt, Frankfurt, Deutschland; 14 Geriatrisches Zentrum und Netzwerk Altersmedizin, Agaplesion Bethanien Krankenhaus Heidelberg, Universität Heidelberg, Heidelberg, Deutschland

**Keywords:** Altern, Geriatrie, SARS-CoV-2, Pandemie, Rehabilitation, Aging, Geriatrics, SARS-CoV-2, Pandemia, Rehabilitation

## Abstract

Schwerwiegend verlaufende COVID-19-Erkrankungen betreffen vorwiegend die ältere Bevölkerung. Die Mortalität der hospitalisierten COVID-19-Patienten im Alter über 80 Jahre liegt international bei bis zu 54 %. Daher ist ein Blick auf die Erkrankung aus geriatrischer Perspektive erforderlich. Diagnostik und Therapie der COVID-19-Erkrankung unterscheiden sich bei den älteren Patienten nicht grundsätzlich von der bei jüngeren Patienten. Allerdings ist bei Patienten im hohen Alter gehäuft mit einer atypischen Symptomatik zu rechnen. Der Rehabilitationsbedarf nach durchgemachter Infektion ist bei älteren COVID-19-Patienten deutlich höher als bei jüngeren Patienten. Paradoxerweise steht der Notwendigkeit vermehrter Rehabilitationsleistungen jedoch ein sinkendes Angebot geriatrischer Rehabilitationsmöglichkeiten gegenüber, da viele Abteilungen entweder geschlossen oder deren Behandlungskapazitäten reduziert wurden. Generell sollten Quarantäne- und Isolationsmaßnahmen in der älteren Bevölkerung verstärkt auf ihre Verhältnismäßigkeit überprüft werden, da die gesundheitlichen und emotionalen Auswirkungen gravierend sein können. Angesichts der ungünstigen Prognose bei hochaltrigen COVID-19-Patienten kommt der Berücksichtigung des Patientenwillens eine besondere Bedeutung zu. Daher sollten Angehörige und Ärzte sich frühzeitig, d. h. möglichst bereits vor dem Auftreten einer Infektion, bemühen, diesen zu eruieren und angemessen zu dokumentieren. Erfreulicherweise lassen die bisherigen Daten hoffen, dass die Impfung mit den in Deutschland zugelassenen mRNA-Impfstoffen gegen SARS-CoV‑2 auch im hohen Alter gut wirksam ist.

## Abstract (en)

Predominantly the older population is affected by a severe course of COVID-19. The mortality of hospitalized patients with COVID-19 above the age of 80 years is up to 54% in international studies. These observations indicate the necessity to highlight the geriatric perspective on this disease. The diagnostics and treatment of COVID-19 do not differ between younger and older patients but atypical symptoms should be expected more frequently in old age. Older subjects show an increased need for rehabilitation after COVID-19. Paradoxically, increasing rehabilitation demands go along with a reduced availability of geriatric rehabilitation options, the latter being a consequence of closure or downsizing of rehabilitation departments during the pandemic. In general, measures of isolation and quarantine should be diligently balanced as the health and emotional consequences of such measures may be severe in older persons. In light of the poor prognosis of older COVID-19 patients, advanced care planning becomes even more relevant. Caregivers and physicians should be encouraged to compose advanced care directives that also reflect the specific circumstances of COVID-19. Fortunately, current data suggest that the effectiveness of the vaccination with the mRNA-vaccines approved in Germany may be equally high in older compared to younger persons.

## Einleitung

Die aktuelle Coronapandemie stellt sowohl das Gesundheitssystem wie auch die Bevölkerung vor große Herausforderungen. Da die COVID-19-Erkrankung mit schwerem Verlauf vorwiegend die ältere Bevölkerung betrifft und in dieser auch häufig mit schwerwiegenden gesundheitlichen Folgen einhergeht, ist ein Blick auf diese Erkrankung aus geriatrischer Perspektive erforderlich. Warum erkranken besonders ältere Menschen? Welche Besonderheiten weisen Symptomatik und Verlauf im Alter auf? Welche Konsequenzen können infektionsprophylaktische Maßnahmen in der älteren Bevölkerung haben? Die folgende Übersicht wird auf der Basis des gegenwärtigen wissenschaftlichen Kenntnisstandes Antworten auf diese und weitere Fragen geben.

## Biologisches Alter und Frailty

Bereits in der Frühphase der Pandemie zeigte sich, dass bevorzugt ältere Personen einen schwereren Krankheitsverlauf und eine erhöhte Mortalität aufwiesen [18, 19]. Im weiteren Verlauf wurde jedoch deutlich, dass dem chronologischen Alter aufgrund der Heterogenität der älteren Bevölkerung nur eine eingeschränkte prognostische Bedeutung zukommt. Neben der individuell vorhandenen Komorbidität ist in diesem Zusammenhang das biologische Alter von besonderer Relevanz. Mehrere Schlüsselprozesse des Alterns interagieren mit dem Verlauf einer SARS-CoV-2-Infektion und können ihren Verlauf beeinflussen [43, 62]. Als Beispiele von im Kontext der SARS-CoV-2-Infektion bedeutsamen Alterungsprozessen sind zu nennen:

- zelluläre Seneszenz, bes. im Bereich des Immunsystems,
- Inflammaging,
- Instabilität des Genoms,
- mitochondriale Dysfunktion,
- reduzierte Telomerlänge,
- epigenetische Veränderungen,
- gestörte Autophagie.

Unter diesen Phänomenen scheint der zellulären Seneszenz eine besondere Bedeutung zuzukommen, da sie sowohl im Bereich des Immunsystems als auch im respiratorischen Epithel und Gefäßendothel den Verlauf einer SARS-CoV-2-Infektion beeinflussen kann. Der geschlechtsspezifisch unterschiedlich schwere Verlauf der Infektion, mit einer deutlich schlechteren Prognose bei Männern, dürfte zumindest teilweise durch geschlechtsspezifische Alterungsvorgänge im Bereich der Zellen des Immunsystems bedingt sein.

Leider besteht gegenwärtig noch keine Möglichkeit, die zelluläre Seneszenz oder spezifisch die Immunseneszenz anhand eines klinisch nutzbaren Tests zu erfassen. Zur Einschätzung des biologischen Alters kann jedoch das Frailty-Konzept als ein Ansatz verstanden werden, in der klinischen Routine Morbiditätsfaktoren und biologisches Alter in einem Erfassungsinstrument zu integrieren. Mehrere Studien belegen, dass die Erfassung der Frailty sowohl dem chronologischen Alter als auch dem Vorliegen einer Multimorbidität als prädiktiver Faktor überlegen ist [37, 43, 62]. Während in der Studie von Hewitt et al. die Clinical Frailty Scale (CFS) Anwendung fand, war dies bei Aw et al. der in Großbritannien entwickelte elektronische Frailty-Score, welcher anhand von NHS-Routinedaten aus dem hausärztlichen Bereich erstellt wird. Bezüglich der Anwendung der CFS ist es wichtig zu beachten, dass sich diese auf den Zustand vor Beginn der akuten Erkrankung beziehen muss. In mehreren Studien wurde ein Abstand von 2 bis 3 Wochen vor Erkrankungsbeginn zugrunde gelegt. Die geringste Überlebensrate fand sich bei geriatrischen Patienten, falls eine Frailty in Kombination mit einer Multimorbidität vorlag (>2 Erkrankungen). Das Vorliegen einer Frailty und/oder einer Multimorbidität im Kontext einer COVID-19-Erkrankung hat somit wesentliche Bedeutung für die Prognose älterer Patienten. Gleichwohl dürfen die im individuellen Fall vorzuhaltenden diagnostisch-therapeutischen Maßnahmen nicht allein auf dieser Basis festgelegt werden. Die Therapieplanung erfordert eine umfassende Analyse der individuellen Situation unter Berücksichtigung des (mutmaßlichen) Patientenwillens.

## Behandlungsergebnisse und Prognose

In einer großen multizentrischen Studie während der ersten Pandemiewelle in Deutschland wurden 17 % der hospitalisierten Patienten aller Altersgruppen maschinell beatmet [28]. Die Krankenhaussterblichkeit der beatmeten Patienten im Alter von 80 Jahren und älter lag dabei bei 72 %. Auch die Mortalität in der Gesamtgruppe der hospitalisierten COVID-19-Patienten ist ebenfalls besorgniserregend hoch. Bei 5700 hospitalisierten COVID-19-Patienten in New York fand sich in den Altersgruppen 60 bis 69, 70 bis 79, 80 bis 89 und > 89 Jahre eine Krankenhaussterblichkeit von 15,8, 32,2, 54,3 und 52,3 % [55]. Diese ausgesprochen hohe Sterblichkeit der hospitalisierten Patienten in New York muss sicherlich wegen der zeitweisen Überlastung des dortigen Gesundheitssystems und der Besonderheiten der US-amerikanischen Krankenversicherung relativiert werden. In einer Metaanalyse altersstratifizierter Registerdaten aus China, Italien, Spanien, United Kingdom und New York finden sich mittlere Mortalitätsraten von 0,3 %, 0,5 %, 1,1 %, 3,0 %, 9,5 %, 22,8 % und 29,6 % in den Altersgruppen < 29, 30 bis 39, 40 bis 49, 50 bis 59, 60 bis 69, 79 bis 79 und ≥ 80 Jahre [6]. Zu betonen ist, dass die Daten dieser Studie die Sterblichkeit aller registrierten COVID-19-Erkrankungen angeben, also nicht nur der hospitalisierten Patienten. Eine deutlich erhöhte Sterblichkeit in der älteren Bevölkerung ließ sich auch in vielen anderen Studien und Ländern, Deutschland eingeschlossen, nachweisen [32, 34]. In der Gruppe der Pflegeheimbewohner fand sich in einer US-amerikanischen Studie in der Gesamtgruppe der SARS-CoV-2-Infizierten eine 30-Tages-Mortalität von 21 % ohne Berücksichtigung des Alters; bei den ≥ 80-Jährigen lag die 30-Tages-Mortalität bei 30 % [46]. Dies deckt sich etwa mit den nichtalterstratifizierten Daten des Robert Koch-Instituts für deutsche Pflegeheimbewohner mit COVID-19, von denen 16,7 % verstorben sind [59].

Allerdings werden bei all diesen Angaben demografische Unterschiede, Meldefehler und asymptomatische nichtdiagnostizierte Infektionen nicht berücksichtigt. Modellrechnungen mit Berücksichtigung dieser Faktoren kommen in der Gruppe der Personen ≥ 80 Jahre zu einer Case Fatality Rate (CFR) von 13,4 % und einer Infection Fatality Rate (IFR) von 9,3 % [63]. Dies bedeutet, dass 13,4 % der symptomatischen Infizierten ≥ 80 Jahre und 9,3 % aller infizierten Menschen ≥ 80 Jahre an dieser Erkrankung versterben. Dies deckt sich in etwa mit den aktuellen Daten des Robert-Koch-Instituts. Aus diesen lässt sich aktuell eine Mortalität von 17,6 % in der Gesamtgruppe positiv-getesteter Menschen ≥ 80 Jahre (Männer 24,0 %, Frauen 14,4 %) errechnen [58]. Abgesehen von den aktuellen Kumulativdaten des Robert Koch-Instituts muss bei allen oben genannten Studien berücksichtigt werden, dass es sich überwiegend um Daten aus der ersten Erkrankungswelle handelt. Inzwischen hat das Wissen über diese neue Erkrankung zugenommen und sowohl das Pandemiemanagement als auch die Diagnostik und Therapie der Erkrankung wurden optimiert, sodass gegenwärtig zumindest eine leichte Verbesserung der CFR zu erwarten wäre.

Für den Zweck von Aufklärungsgesprächen könnte man daher grob zusammenfassen, dass in der Gruppe der COVID-19-Patienten ≥ 80 Jahre etwa 15 % der Gesamtgruppe, 25 % der hospitalisierten und 70 % der beatmeten Patienten mit oder an der COVID-19-Erkrankung versterben. Je nach Geschlecht, Komorbidität und Frailty-Status müssten diese Zahlen allerdings nach oben oder unten adjustiert werden. Hierbei konnte die Studie von Ho et al. darstellen, dass Patienten ≥ 75 Jahre gegenüber den < 65-jährigen eine 4‑fach erhöhte Mortalität aufwiesen, wenn keine zusätzlichen Risikofaktoren vorlagen. Waren die typischen Risikofaktoren vorhanden, so war das Risiko um den Faktor 13 gesteigert [24].

Berücksichtigt werden muss auch, dass das Behandlungsergebnis der überlebenden geriatrischen Patienten insbesondere nach einer intensivmedizinischen Behandlung in der Regel mit einer Verschlechterung der Funktionalität und Selbstständigkeit verbunden ist. Daher ist die frühzeitige Einbindung des Geriaters von Bedeutung, um den weiteren, (früh)rehabilitativen Verlauf adäquat steuern zu können.

## Vorausschauende Versorgungsplanung

Angesichts der häufig schweren Krankheitsverläufe in der älteren Bevölkerung sollte gerade bei älteren Personen eine vorausschauende Versorgungsplanung („advance care planning“, ACP) stattfinden. Zu diesem Zweck sollten sich ältere und multimorbide Patienten idealerweise zusammen mit ihrem betreuenden Hausarzt mit diesem Thema befassen. Auch sollten im Falle einer Hospitalisierung aufgrund einer COVID-19-Erkrankung bereits in der Aufnahmeroutine nach einer Patientenverfügung, Vorsorgevollmacht und den Wünschen bezüglich der Invasivität der Behandlung bei klinischer Verschlechterung gefragt und die Ergebnisse entsprechend dokumentiert werden. In der aktuellen Situation der Coronapandemie sollte konkret geklärt werden, ob eine akutmedizinische Therapie im Krankenhaus gewünscht ist und inwiefern ggf. eine nichtinvasive oder invasive Beatmung erfolgen sollen. Dazu muss das in einem hohen Prozentsatz ungünstige Behandlungsergebnis bei hochaltrigen Patienten, bei denen im Rahmen einer COVID-19-Erkrankung eine invasive Beatmung notwendig ist, offengelegt werden, damit ältere Menschen, ggf. zusammen mit den Angehörigen, die Entscheidung gut abwägen können.

## Therapieansätze, inklusive intensivmedizinischer Versorgung

Bezüglich detaillierter Behandlungsempfehlungen sei auf die aktuelle S2k-Leitlinie verwiesen [30]. Der folgende Abschnitt soll lediglich einen Überblick über die wesentlichen aktuellen Therapiestrategien vermitteln. Die Indikation zur Krankenhausaufnahme von COVID-19-Patienten erfolgt nach klinischen Kriterien unter Berücksichtigung von Alter, Komorbiditäten, Atemfrequenz und Sauerstoffsättigung unter Berücksichtigung des Patientenwillens.

In der Frühphase der Erkrankung (≤10 Tage nach Symptombeginn) kann bei nichtbeatmeten Patienten mit COVID-19-Pneumonie die Gabe von Remdesivir erwogen werden [4]. Andere antivirale Substanzen werden außerhalb klinischer Studien nicht empfohlen. Bei Patienten mit schwerer (S_p_O_2_ < 90 %, Atemfrequenz > 30/min) oder kritischer COVID-19-Erkrankung (z. B. ARDS, Sepsis, Beatmung) ist eine Therapie mit Dexamethason indiziert. Die Indikation zur prophylaktischen Antikoagulation ist bei hospitalisierten Patienten aufgrund der COVID-19-assoziierten Koagulopathie (CAC) und der pulmonalen intravaskulären Koagulopathie (PIC) frühzeitig und großzügig zu stellen. Bei Intensivpatienten sollte eine intensivierte Antikoagulation erwogen werden. Für eine Antibiotikatherapie gibt es keine Indikation, solange keine Hinweise auf eine bakterielle Begleit- oder Superinfektion bestehen.

Bei progredienter Verschlechterung des Gasaustausches sind oftmals eine Sauerstoffgabe, eine High-Flow-Sauerstofftherapie und eine (nichtinvasive/invasive) Beatmung notwendig. Bezüglich der Oxygenierung sollten eine periphere Sauerstoffsättigung ≥ 90 % (bei COPD-Patienten > 88 %) bzw. ein O_2_-Partialdruck > 55 mm Hg angestrebt werden. Die extrakorporale Membranoxygenierung (ECMO) kam bisher in der Gruppe der geriatrischen Patienten kaum zum Einsatz [2], da in vielen Ländern und Zentren ein Alter über 70 Jahre, schwere Komorbiditäten und eine zu erwartende lange Beatmungsdauer als Kontraindikation für dieses Verfahren gelten.

Auch bei den alten Patienten sind die Schwere und die Anzahl der Organversagen, gemessen am Sequential Organ Failure Assessment (SOFA) Score ein bedeutsamer Prädiktor für die Sterblichkeit [67]. Zur schnellen und praktikablen Einschätzung der individuellen Prognose eines alten Patienten erlangt die CFS auch für die Intensivpatienten zunehmende Bedeutung [27, 41]. Nicht nur die Entscheidung über den Beginn einer intensivmedizinischen Behandlung muss sorgfältig abgewogen werden, auch die Weiterführung bedarf der regelmäßigen Evaluation [56]. Wenn die Prognose zum Ausgangszeitpunkt nicht beurteilbar ist, ist ein zeitlich begrenzter Therapieversuch („time-limited trial“, TLT) durchaus sinnvoll, um ohne Zeitdruck eine fundierte Entscheidung herbeizuführen [70].

Vor dem Hintergrund der aktuellen Pandemie, deren Verlauf gegenwärtig weiterhin nicht abschätzbar ist, ist es sinnvoll, ein Konzept für eine Entscheidungshilfe bei knappen Ressourcen bereits im Vorfeld einer möglichen Überlastung des Gesundheitssystems zu erarbeiten. Die CFS allein ist dabei für die Indikationsstellung für oder gegen eine intensivmedizinische Therapie unzureichend. In diesem Kontext kam es zu intensiven Diskussionen über die Zuteilung intensivmedizinischer Ressourcen im Zusammenhang mit der COVID-19-Pandemie [36]. Der aus der Katastrophenmedizin bekannte Begriff der Triagierung sollte im Zusammenhang mit der COVID-Pandemie aus ethisch-moralischer Sicht nicht benutzt werden. Hier scheint hingegen der Begriff der „individuellen Priorisierung“ geeigneter. Die Initiierung und Durchführung einer intensivmedizinischen Behandlung von geriatrischen COVID-Patienten sollte – wie bei allen Patienten – unter Berücksichtigung ethischer Prinzipien (Fürsorge, Selbstbestimmung, Nicht-Schaden und Gerechtigkeit) erfolgen. Die Entscheidung zur Intensivtherapie sollte auch in COVID-Pandemie-Zeiten auf den beiden Säulen der medizinischen Indikation und des Patientenwillens basieren. Stehen die intensivmedizinischen Ressourcen nicht mehr für alle Patienten zur Verfügung, so sollten sowohl COVID-Patienten als auch Nicht-COVID-Patienten im Rahmen der individuellen Priorisierung stets gleichwertig betrachtet werden.

## Die neurologischen Manifestationen

Viele geriatrische Patienten leiden an neurologischen Vorerkrankungen oder entwickeln diese im Rahmen einer COVID-19-Erkrankung, weshalb die neurologischen Manifestationen der SARS-CoV-2-Infektion hier gesondert Erwähnung finden. Es gibt keine Hinweise darauf, dass Patienten mit neurologischen Grunderkrankungen häufiger an COVID-19 erkranken als solche ohne neurologische Erkrankungen. Allerdings haben COVID-19-Patienten mit neurologischen Vorerkrankungen ein um 38 % höheres Risiko, im Krankenhaus zu versterben [16]. Eine SARS-CoV-2-Infektion kann spezifisch das Zentralnervensystem (ZNS) und das periphere Nervensystem affizieren, wobei die initiale Riech- und Geschmacksstörung bereits als Hinweis auf den Neurotropismus des Virus zu werten ist. SARS-CoV‑2 kann, unabhängig von der Schwere der Atemwegsinfektion oder anderer Organbeteiligungen, zu verschiedenen neurologischen Manifestationen und Komplikationen führen. Aktuell werden vier Pathomechanismen beschrieben, über die das Nervensystem geschädigt werden kann.

Zum einen kann die virale Schädigung der Nervenzellen über einen direkten Kontakt zwischen Virus und Neuron mit Virusreplikation im Neuron verursacht werden (z. B. analog der Herpes-simplex-Virus-Typ 1-Enzephalitis). Bei Anosmie wird in diesem Zusammenhang eine Bulbus-olfactorius-Schädigung diskutiert, die dabei auch als Eintrittspforte in das ZNS dienen könnte [17]. Über diesen Mechanismus könnte das Virus in das ZNS gelangen und dort entlang neuroanatomischer Strukturen in verschiedene Hirnregionen gelangen [39]. Die bisher publizierten Liquoranalysen bei mit SARS-CoV-2-infizierten Patienten konnten allerdings nur sehr selten SARS-CoV‑2 per Polymerase-Kettenreaktion (PCR) im Liquor detektieren oder die Synthese intrathekaler Antikörper gegen SARS-CoV‑2 im Liquor nachweisen [15, 40, 44]. Eine negative Liquor-PCR schließt (wie bei anderen Virusinfektionen) das Vorhandensein von Coronaviren im Hirngewebe jedoch nicht aus [44].

Der zweite Schädigungsmechanismus beinhaltet eine überschießende Immunantwort in Form einer toxischen proinflammatorischen Zytokinausschüttung [23], welche für die akute respiratorische Insuffizienz mit häufiger Beatmungspflichtigkeit und die hohe Letalität verantwortlich gemacht wird und vermutlich ebenso zu zentralen Nebenwirkungen durch eine Mikroglia-Inflammasom-Aktivierung führt [23].

Ein dritter Schädigungsmechanismus beinhaltet eine pathologische Immunantwort mit Kreuzreaktivität wie z. B. beim Guillain-Barré(GBS)- oder Miller-Fisher-Syndrom. Dieser Mechanismus ist bereits bei verschiedenen anderen Erregern bekannt [48].

Der vierte Schädigungsmechanismus beinhaltet vaskuläre Effekte, einschließlich der oben genannten Koagulopathie, wobei sich vermutlich dieser und der hyperinflammatorische Schädigungsmechanismus häufig überlappen.

In der akuten Phase der COVID-19-Erkrankung entwickeln 36 % der Erkrankten neurologische Symptome, 25 % im Sinne einer ZNS-Beteiligung [23]. Das neurologische Symptomspektrum reicht von Riech- und Geschmacksstörungen über Schlaganfälle, Epilepsie und Lähmungen bis hin zu Delir und der multiplen Sklerose ähnlichen Bildern. Die Vielzahl an Veröffentlichungen von Fallserien und Studien führte daher zur Bezeichnung „Neuro-COVID“. Bis zu 30 % der Betroffenen sind im Sinne des Post-COVID-Syndroms auch nach Abklingen der akuten Erkrankung nicht beschwerdefrei. Im Vordergrund stehen dabei psychomotorische und kognitive Funktionsstörungen wie Müdigkeit, Fatigue, mangelnde Konzentrationsfähigkeit und reduzierte Belastbarkeit. In einigen Fällen bleiben aber auch weitere neurologische Symptome und Ausfälle zurück [22]. Diesen Funktionsstörungen gilt es, insbesondere im Alter mit einem rehabilitativen Ansatz zu begegnen, um einer Akzeleration geriatrischer Funktionseinschränkungen in Bezug auf Alltagsaktivitäten und Lebensqualität entgegenzuwirken. Es liegt nahe, dass bereits durch neurodegenerative und zerebrovaskuläre Vorerkrankungen vorbestehende psychomotorische und kognitive Funktionsstörungen im Alter durch eine COVID-19-Erkrankung kritisch verschlimmert werden können [23].

In einer Verlaufsstudie fanden die Autoren eine Prävalenz neurologischer Symptome von mehr als 80 % bei Krankenhausaufnahme [33]. Mehr als 40 % der Patienten boten bereits zu Beginn der Erkrankung Beschwerden vonseiten des Nervensystems. Besonders häufig waren Muskelschmerzen (45 %), Kopfschmerzen (38 %), Enzephalopathie (32 %), Benommenheit (30 %), Dysgeusie (16 %) und Anosmie (12 %). Eine Enzephalopathie war unabhängig von anderen Faktoren mit einem deutlich schlechteren funktionellen Behandlungsergebnis und einer höheren Mortalität innerhalb von 30 Tagen nach Aufnahme assoziiert [33]. In einer kleineren Studie mit 30 Patienten war das häufigste neurologische Symptom ein auffälliger kognitiver Status bei 33 %, gefolgt von Paresen (30 %), Bewusstseinsstörungen (23 %), Hypo‑/Areflexie (30 %), Hyp‑/Anosmie oder Hypo‑/Ageusie (20 %) und epileptischen Anfällen (17 %). Die häufigsten neurologischen Diagnosen beinhalteten Enzephalopathie (37 %), zerebrovaskuläre Ereignisse (17 %) sowie (Poly)Neuropathien (30 %), davon ein Patient mit Miller-Fisher-Syndrom und 2 mit GBS [44].

Bei älteren Menschen kann ein Delir erstes und einziges Symptom einer COVID-19-Erkrankung sein. Patienten mit einem unklaren Verwirrtheitszustand in der Notaufnahme sollten daher immer auf SARS-CoV‑2 getestet werden [29]. In einer Studie, in die ältere Menschen mit einem Durchschnittsalter von 78 Jahren eingeschlossen wurden, war das Delir das sechsthäufigste Symptom einer COVID-19-Erkrankung, und bei 17 % der Delirpatienten war das Delir primäres Symptom von COVID-19.

Die Diagnostik und Therapie der neurologischen Manifestationen bei COVID-19 folgen den Empfehlungen der Leitlinien der Deutschen Gesellschaft für Neurologie [5, 45].

## Die Rolle der Ernährung

Aufgrund der hohen Prävalenz der Malnutrition im Alter und ihres negativen Einflusses auf das Immunsystem stellt sich im Kontext der Coronapandemie die Frage, inwieweit der Ernährungsstatus für die Prognose und Therapie der Erkrankung von Bedeutung sein könnte. Dabei erhöht das Vorliegen einer Mangelernährung eindeutig das Risiko für einen schweren bzw. tödlichen Verlauf einer COVID-19-Erkrankung [54]. Dass eine Mangelernährung auch das Risiko für das Auftreten einer SARS-CoV-2-Infektion erhöht, wird vermutet, ist aber bisher nicht bewiesen. Je nach verwendetem Screeninginstrument und Studienpopulation finden sich bei älteren COVID-19-Patienten unterschiedliche Prävalenzen für Mangelernährung. Abhängig vom verwendeten Screening-Instrument liegt die Prävalenz der Mangelernährung bei etwa 50 % [66]. Bei Anwendung der neuen Diagnosekriterien der Global Leadership Initiative on Malnutrition (GLIM) ist bei 42–50 % der COVID-19-Patienten eine Malnutrition zu diagnostizieren [3, 50]. In einer italienischen Studie verloren 29 % der COVID-19-Patienten während des Krankheitsverlaufs mehr als 5 % ihres Körpergewichts [12]. Ein Gewichtsverlust war dabei mit einer längeren Erkrankungs- und Hospitalisierungsdauer assoziiert [12].

Neben dem COVID-assoziierten Verlust der Geruchs- und Geschmackswahrnehmung spielt die krankheitsassoziierte Hyperinflammation eine besondere Rolle in der Pathogenese der Malnutrition. Schon in der Vergangenheit ließ sich bei geriatrischen Patienten nachweisen, dass Inflammation mit einem Verlust des Appetits und einer unzureichenden Nahrungsaufnahme assoziiert war [42, 53, 65]. Da sowohl eine vorbestehende Malnutrition wie auch eine im Rahmen einer COVID-19-Erkrankung entstandene Malnutrition von großer Bedeutung für den weiteren Krankheitsverlauf sind, sollte bei allen COVID-19-Patienten ein Screening auf Malnutrition erfolgen. Silva et al. konnten für alle gängigen Screeninginstrumente auch bei COVID-19-Erkrankung eine hohe Sensitivität nachweisen [66], sodass in der Regel das Screeninginstrument genutzt werden kann, welches in der jeweiligen Einrichtung bereits etabliert ist. Es ist zudem wichtig, das Screening wöchentlich zu wiederholen. Dies gilt auch für Patienten, deren COVID-19-Infektion ambulant oder im Pflegeheim behandelt wird. Im Falle einer unzureichenden Ernährung sollte gemäß den aktuellen Leitlinien zu Ernährung und Flüssigkeitsversorgung geriatrischer Patienten gehandelt werden, um eine ausreichende Energie- und Proteinzufuhr zu erzielen [71].

Vor dem Hintergrund der häufigen Malnutrition stellt sich auch die Frage, ob und welche Mikronährstoffe für Menschen mit einer COVID-19-Erkrankung von besonderer Bedeutung sein könnten. Unstrittig ist, dass Menschen mit erniedrigtem Vitamin-D-Spiegel eine höhere Wahrscheinlichkeit für eine COVID-19-Infektion und für einen schwereren Verlauf aufweisen [35]. Da niedrige Vitamin-D-Spiegel allerdings auch stark mit Multimorbidität und eingeschränkter Mobilität assoziiert sind, bleibt hier die Kausalbeziehung zunächst offen. Randomisierte Interventionsstudien sind hierzu bisher nicht verfügbar. Ähnliches gilt für die Tatsache, dass die Magnesium- und Kalziumspiegel im Rahmen einer COVID-19-Infektion abfallen. In einer Pilotstudie konnte durch den Vergleich einer frühen und späteren Kohorte von COVID-19-Erkrankten gezeigt werden, dass die Gabe von Vitamin D, Magnesium und Vitamin B_12_ die Rate der Patienten mit Sauerstoffbedarf signifikant senken konnte [68]. Der Ausgleich eines Vitamin-D-Mangels ist während der Pandemie daher dringend anzuraten.

Insgesamt muss eine adäquate Ernährungstherapie von der Akutphase bis zur Rehabilitation in die Therapie der COVID-19-Erkrankung integriert sein [1].

## Körperliche und psychische Auswirkungen von Quarantäne und Isolationsmaßnahmen

Soziale Isolation, wie sie z. B. aus Kontakt- und Ausgangsbeschränkungen oder Quarantänemaßnahmen resultieren kann, erhöht die Inzidenz einer Vielzahl chronischer altersassoziierter Erkrankungen, verschlechtert deren Verlauf und trägt damit zur Übersterblichkeit alter Menschen bei [47, 51]. Entsprechende Zusammenhänge wurden für die arterielle Hypertonie, die koronare Herzerkrankung, die Herzinsuffizienz, den Diabetes mellitus und die Demenz beschrieben und sind heute durch zahlreiche prospektive Studien empirisch abgesichert. Soziale Isolation und Einsamkeit stellen darüber hinaus unabhängige Risikofaktoren für zerebrovaskuläre und kardiale Akutereignisse dar [13]. Indem sie das Immunsystem schwächen, entfalten sie in puncto Infektionsschutz eine geradezu paradoxe Wirkung [38]. Isolationsbedingte körperliche Inaktivität sowie ein ungünstiges Ernährungsverhalten mit konsekutiver Mangelernährung werden als vermittelnde Faktoren diskutiert [7, 31, 49], denn diese begünstigen Sarkopenie und eine reduzierte kardiorespiratorische Fitness, was wiederum eine Zunahme von Immobilität und Sturzereignissen bei sozial isolierten alten Menschen erklärt [21]. Die Compliance bei der Medikamenteneinnahme nimmt ab, wodurch der Verlauf und die Prognose vorbestehender Erkrankungen zusätzlich verschlechtert werden. Entsprechend steigt bei sozial isolierten alten Menschen das Risiko für Krankenhauseinweisungen [13], Pflegebedürftigkeit und Heimunterbringung [61]. Soziale Isolation und Einsamkeit stellen darüber hinaus psychosoziale Stressoren dar, die Wohlbefinden und Lebensqualität erheblich beeinträchtigen. Ein Mangel an wertschätzenden, positiv anregenden zwischenmenschlichen Erfahrungen begünstigt gerade auch bei alten, körperlich beeinträchtigten Menschen die Entwicklung von apathischen und depressiven Syndromen [8, 13]. Hierdurch steigt das Suizidrisiko, das in dieser Bevölkerungsgruppe im Vergleich zur Allgemeinbevölkerung ohnehin deutlich erhöht ist. Angst- und Anpassungsstörungen sowie ein riskanter bzw. missbräuchlicher Alkoholkonsum sind weitere psychische Komorbiditäten, die bei älteren, isoliert lebenden Menschen vermehrt beobachtet werden [13]. Soziale Isolation ist darüber hinaus ein etablierter Risikofaktor für kognitive Beeinträchtigung bis hin zur Demenz [8]. Bereits vorbestehende kognitive Defizite können durch die Reduzierung sozialer Kontakte aggraviert werden. Diese Zusammenhänge werden einerseits über die oben beschriebenen somatischen und verhaltensbezogenen Demenzrisikofaktoren (arterielle Hypertonie, Diabetes mellitus, Bewegungsmangel, Mangelernährung, zerebrovaskuläre Erkrankung) vermittelt. Andererseits führt soziale Isolation zu einem Mangel an kognitiv-stimulierenden Alltagserfahrungen, die gerade bei alten, multimorbiden Patienten für den Erhalt der kognitiven Kompetenzen von großer Bedeutung sind.

Vor Ausbruch der SARS-CoV-2-Pandemie konzentrierte sich die Forschung überwiegend auf die Untersuchung mittel- bis langfristiger Effekte sozialer Isolation unter Alltagsbedingungen. Aussagekräftige Studien zu den gesundheitlichen Folgen kurzer, aber intensiver Isolationserfahrungen, wie sie z. B. durch pandemiebedingte Quarantäneverfügungen oder Besuchsverbote in Pflegeeinrichtungen verursacht werden, waren vergleichsweise rar. Untersuchungen, die während der zurückliegenden SARS-CoV-1- und MERS-Pandemien durchgeführt wurden, wiesen bei Menschen mit Quarantäne-Erfahrungen aller Altersgruppen eine bedeutsame Zunahme von akuten Stresssymptomen, Schlafstörungen, Angstsymptomen, Depressivität und posttraumatischen Belastungsstörungen nach [60]. Diese psychischen Störungen stellten sich bereits nach relativ kurzer Quarantänedauer ein und waren bei gut einem Viertel der Betroffenen noch über ein halbes Jahr nach Beendigung der Quarantäne nachweisbar. Telefonische bzw. postalische Erhebungen an umfangreichen Stichproben alter Menschen, die während der ersten Welle der SARS-CoV-2-Pandemie in der Schweiz, in Hong-Kong und London durchgeführt wurden, bestätigen diese Befunde [57, 64, 72]: Insbesondere alleinlebende und multimorbide alte Menschen berichteten unter den Bedingungen des Lockdowns über eine Zunahme von Einsamkeit, Angstsymptomen, Depressivität und Schlafstörungen. Darüber hinaus wurden medizinische Kontrolltermine nicht in dem Maße wahrgenommen wie vorgesehen.

Diese Ergebnisse weisen darauf hin, dass bereits relativ kurze Phasen intensivierter sozialer Isolation den Gesundheitszustand alter Menschen verschlechtern können, insbesondere dann, wenn diese multimorbide sind und nicht über ausreichende soziale Netzwerke verfügen. Der psychischen und sozialen Situation dieser Bevölkerungsgruppen sollte daher gerade unter Pandemiebedingungen besondere Aufmerksamkeit geschenkt werden. Infektionsschutzmaßnahmen, die soziale Isolation fördern, sollten so kurz wie möglich gehalten und kontinuierlich hinsichtlich ihrer Verhältnismäßigkeit hinterfragt werden.

## Rehabilitationsbedarf

Mehrere Arbeiten belegen, dass es bei älteren Patienten im Rahmen einer COVID-19-Erkrankung in einem relevanten Prozentsatz zu einer Verschlechterung des funktionellen Status und der Fähigkeit zur Selbstversorgung kommt. So war in einer britischen Studie mit mehr als 800 Teilnehmern zum Zeitpunkt der Krankenhausentlassung bei 25 % der Patienten der verstärkte Einsatz ambulanter Hilfen erforderlich [69]. In diesem Kollektiv erwies sich die CFS als ein zuverlässiger Prädiktor einer zu erwartenden funktionellen Verschlechterung. Die CFS wurde bei Krankenhausaufnahme erfasst und bezog sich auf den Status 2 Wochen vor dem akuten Geschehen. Die Autoren dieser Arbeit fordern den routinemäßigen Einsatz der CFS bei der stationären Aufnahme von COVID-19-Patienten, um den individuellen Rehabilitationsbedarf frühzeitig abschätzen zu können. Da insbesondere ältere Personen mit bereits vorbestehender Multimorbidität und Frailty von schweren Verläufen der COVID-Erkrankung betroffen sind, muss ein zukünftig adäquates rehabilitatives Angebot diese Ausgangssituation stärker berücksichtigen.

Die in einem hohen Prozentsatz protrahiert verlaufende COVID-19-Erkrankung, die dann auch als „long COVID“ bezeichnet wird, betrifft oftmals mehrere Organsysteme mit einem weiten Spektrum möglicher funktioneller Beeinträchtigungen, die in Tab. 1 zusammengestellt sind [14]. Die in der aktuellen zweiten Erkrankungswelle zu beobachtende große Zahl an schwer erkrankten älteren COVID-19-Patienten wird einen rasch anwachsenden Rehabilitationsbedarf in dieser speziellen Untergruppe verursachen.

**Table 1:** 

Sarkopenie als Folge von Immobilität, Hyperinflammation und Malnutrition
Respiratorische Partialinsuffizienz
Herzinsuffizienz als Folge einer Myokarditis
Kognitive Beeinträchtigung als Folge eines Delirs oder Schlaganfalls
Neuro- und Myopathien
Fatigue
Posttraumatische Belastungsstörung (PTSD)

Dem im Rahmen der Pandemie deutlich gesteigerten Bedarf an Rehabilitationsleistungen steht in vielen Regionen ein sinkendes Angebot gegenüber. Vielerorts wurden rehabilitative Abteilungen geschlossen, um deren Personal in der Akutmedizin zur Versorgung von COVID-19-Patienten einzusetzen. Andere Rehabilitationsabteilungen mussten aufgrund von Ausbrüchen zumindest temporär ihren Betrieb einstellen. Oftmals gestaltete sich in der Folge deren Wiedereröffnung aufgrund von Erkrankungsfällen unter dem ärztlichen, therapeutischen und pflegerischen Personal schwierig. Grund et al. bezeichneten den Umstand eines stark erhöhten Rehabilitationsbedarfs bei sinkendem Angebot treffend als Rehabilitationsparadox [20]. Die Rehabilitation älterer COVID-Patienten erfordert einen starken interdisziplinären Ansatz, welcher das Vorhalten einer Vielzahl von Interventionen einschließt [10]. Diese sind in Tab. 2 gelistet.

**Table 2:** 

Aerobes Training
Krafttraining
Balanceübungen
Training der Alltagsaktivitäten
Respiratorisches Training
Schluck- und Stimmtraining
Ernährungstherapie
Psychologische Betreuung

Ein an die individuellen Bedürfnisse von COVID-19-Patienten angepasstes rehabilitatives Konzept kann die Zusammenarbeit von Ärzten aus verschiedenen Fachgebieten – Geriatrie, Pneumologie, Neurologie und Psychiatrie – erfordern. Entsprechend der lokalen Versorgungssituation sollte die körperliche Aktivierung der Patienten frühzeitig während des Krankheitsverlaufs beginnen, d. h. nach Möglichkeit bereits während der Isolationsphase. Eine Verlegung in einen spezialisierten (Früh‑)Rehabilitationsbereich ist erst dann möglich, wenn der Patient als nicht mehr infektiös eingestuft wird. Aufgrund des oft protrahierten Krankheitsverlaufes und des bei zahlreichen Patienten nach der Akuterkrankung sehr niedrigen funktionellen Ausgangsniveaus bedarf es einer flexiblen Anpassung der Rehabilitationsdauer, die sich an den Fortschritten des Patienten orientieren muss. Die vorhandenen Rahmenbedingungen, wie sie auch in der geriatrischen Rehabilitation vorgesehen sind, entsprechen nicht den aufwendigen Notwendigkeiten. Aus diesen Rahmenbedingungen ergibt sich die Notwendigkeit einer separaten aufwandsentsprechenden Finanzierung, die über der der üblichen geriatrischen Rehabilitation liegen muss. In einer italienischen Arbeit wurde für die Rehabilitation von COVID-19-Patienten eine Verdoppelung des Personalaufwandes ermittelt [25]. Erste Arbeiten weisen darauf hin, dass ein solches Vorgehen für die Post-COVID-19-Patienten eine wesentliche Verbesserung ihres funktionellen Status bedingt [9, 26].

Für den ambulanten Bereich der geriatrischen Rehabilitation haben sich die Rahmenbedingungen in 2020 drastisch verschlechtert. Es wurde keinerlei finanzielle Kompensation für die ambulante und mobile geriatrische Rehabilitation gewährt. Es bleibt abzuwarten, wie viele der Einrichtungen die Pandemie unbeschadet überstehen werden. Inhaltlich sollte ergänzend der Aufbau eines digitalen Angebots zur Nachhaltigkeit aller Rehabilitationsformen entwickelt werden. Dieses muss die besonderen Einschränkungen des älteren Patienten in der Nutzung IT-basierter Angebote berücksichtigen.

## Impfung und Impfbereitschaft

Erfreulicherweise zeigt die ältere Bevölkerung eine sehr hohe Impfbereitschaft. Diese lag in einer Umfrage bei geriatrischen Krankenhauspatienten ≥ 80 Jahre bei 71 % [11]. Im Pflegeheim liegen die Impfquoten nach Auskunft der mobilen Impfteams (MIT) teilweise über 80 %. Während Heimbewohner gegenwärtig von MIT aufgesucht werden, äußerten viele geriatrische Patienten, die im eigenen Haushalt leben, in der oben genannten Umfrage die Sorge, die Impfzentren nicht erreichen zu können und Probleme bei der Terminbeschaffung zu haben. Hier bedarf es dringend einer Anpassung der Strategie. Diese Anpassung ist bis zu dem Zeitpunkt notwendig, ab dem der hausärztliche Sektor über einen für die Hausarztpraxis geeigneten Impfstoff verfügt, denn zwei Drittel der pflegebedürftigen Senioren leben in der eigenen Häuslichkeit und nur ein Drittel im Pflegeheim. Es erscheint realistisch, dass die MIT die meisten Heimbewohner bis Ende März geimpft haben werden. Ob dies bei den über 80-jährigen Menschen in eigener Häuslichkeit gelingt, wird von der Strategie, den Impfstoffmengen und den zugelassenen Impfstoffen abhängen.

Nachdem in der Europäischen Union Anfang Januar der zweite Impfstoff gegen SARS-CoV‑2 zugelassen wurde, stellt sich die Frage, ob diese Impfstoffe vor dem Hintergrund der Immunseneszenz bei älteren Menschen die gleiche Wirksamkeit wie bei jüngeren Menschen aufweisen. Hier lassen die Daten aus der Zulassungsstudie des ersten Impfstoffes zumindest hoffen. In den Probandengruppen im Alter ab 65 und ab 75 Jahren fanden sich die gleiche Wirksamkeit und ein vergleichbares Nebenwirkungsprofil wie bei den jüngeren Probanden [52]. Weiterhin offen ist die Frage, wie lange die Immunität nach einer Impfung tatsächlich anhält, und ob durch die Immunisierung auch die Übertragung der Erkrankung verhindert wird.

## Ausblick

Die COVID-19-Pandemie hat in der ersten und zweiten Welle Schwächen und Stärken des deutschen Gesundheitssystems im Vergleich zu den Nachbarländern offenbart. Während in der ersten Welle mithilfe des Einsatzes der Beschäftigen im Gesundheitswesen, einer ausreichenden Intensivkapazität und eines großen Engagements aller Beteiligten die Versorgungssituation gut bewältigt werden konnte, wurde es während der Sommermonate teilweise versäumt, sich auf die folgende zweite Welle und die Erfordernisse der Impfkampagne gut vorzubereiten. Die Schwierigkeit der Impflogistik wurde ebenso unterschätzt wie die nachlassende Resilienz der Gesundheitsfachberufe. Während der letzten Monate hat die Geriatrie, neben der Intensiv- und Notfallmedizin, eine Hauptlast getragen, wurde aber in der gesundheitspolitischen Diskussion bisher nur wenig gehört. Das Wissen um die Ressourcen und Probleme in der medizinischen und pflegerischen Versorgung älterer Menschen ist unverzichtbar, damit Strategien bedarfsgerecht angepasst werden können. Die Kollateralschäden für die Geriatrie sind erheblich. Viele Mitarbeiter sind selber im Dienst erkrankt, oder manche Kollegen sind verstorben. Im günstigen Fall wird es gelingen, die Pandemie bis zum Herbst zu kontrollieren. Im ungünstigsten Fall werden die Mutationen den Kampf gegen die Pandemie unvorhersehbar verlängern.

COVID-19 wird die gesellschaftliche Diskussion zum Thema Alter und Geriatrie nachhaltig beeinflussen. Es liegt an uns allen, den weiterhin erforderlichen Diskurs mitzugestalten und auf diese Weise die Versorgung der geriatrischen COVID-19-Patienten zukünftig zu verbessern.
